# Effect of grayscale threshold on X-ray computed tomography reconstruction of gas diffusion layers in polymer electrolyte membrane fuel cells

**DOI:** 10.1016/j.heliyon.2024.e29378

**Published:** 2024-04-08

**Authors:** Huarui Li, Tingqiang Qiao, Xiaoyu Ding

**Affiliations:** a Beijing Institute of Technology, School of Mechanical Engineering, 100081, Beijing, China; b AECC Shenyang Engine Research Institute, 110015, Shenyang, China

**Keywords:** Fuel cell, Gas diffusion layer, X-ray computed tomography, Grayscale threshold, Three-dimensional reconstruction

## Abstract

In X-ray computed tomography (CT) reconstructions of gas diffusion layers (GDLs), grayscale threshold selection is a critical issue. Although various selection methods exist, they all have their own drawbacks. This study investigates the influence of grayscale threshold on GDL properties and compares Otsu and porosity-adaptive thresholds. We utilized X-ray CT to reconstruct a Toray carbon paper sample (TGP-H-060) at a resolution of 2 μm. Using reconstructed 3D models generated under different grayscale thresholds, we performed structural analysis, computational fluid dynamics simulation, and compression simulation. We subsequently calculated porosity, tortuosity, permeability, and macroscopic stress-strain relationships, quantitatively analyzing the sensitivity of these parameters to the change of grayscale threshold. The results indicated that small change in the grayscale threshold can significantly impact the transport and mechanical properties of reconstructed GDLs. The difference between Otsu and porosity-adaptive thresholds is notable, and the porosity-adaptive threshold appears to be less accurate than the Otsu threshold.

## 1 Introduction

Polymer electrolyte membrane (PEM) fuel cells are an environmentally friendly, efficient, and clean energy source with broad development potential. A crucial component of the PEM fuel cells is the GDL, which supports functions such as electricity conduction and the transportation of water and reactant gases. Common GDL materials include carbon fiber paper, carbon fiber woven fabric, nonwoven fabric, and carbon black paper, all of which have complex porous structures. These substrate materials facilitate multifaceted physical transport within the pore spaces where mass, electric charge, and energy navigate in all directions under the influence of pressure, concentration, potential, and temperature fields.

The transport and mechanical properties of the GDL significantly influence overall performance of PEM fuel cells and have garnered substantial research attention over the past two decades. In particular, numerical simulations have been widely employed to ascertain the mechanical and transport properties of GDLs. Given the small scale of GDLs (e.g., carbon fiber diameter of around 7 μm) [1], modeling and simulation work typically utilize the volume-averaging (VA) formula. This method, however, tends to smooth out inherent GDL non-uniformities, such as anoxia regions, water nucleation points, and local resistance [2], which limits simulation accuracy. To overcome this limitation, many researchers have turned to X-ray CT for non-destructive three-dimensional (3D) visualization of both sample surface and internal structures, thus acquiring microstructural details of GDLs [[2], [3], [4], [5], [6], [7], [8], [9], [10], [11]]. X-ray CT enables high-resolution reconstruction of GDLs, as reported by Rama [4], who studied the anisotropic permeability of a carbon cloth utilizing X-ray CT at a resolution of 1.7 μm. Similar studies on carbon fiber papers typically have a resolution of approximately 2 μm [2,5,[9], [10], [11]].

Generating a 3D model from original CT images involves complex image processing and binarization. The original CT image is a grayscale one, where a higher grayscale value signifies greater density. Given that different material components (such as carbon and air) vary in density, we can theoretically calculate the spatial distribution of different material components based on grayscale information from CT images, thereby revealing the internal structure of the measured object. A crucial step in this process is determination of the grayscale threshold that distinguishes different material components. Inaccurate grayscale thresholds can distort GDL structures, which can subsequently affect the accuracy of the simulation analysis. Unfortunately, this issue has largely been overlooked in previous research, making it the focal point of the current study.

Various methods are available to determine CT grayscale thresholds, including the Otsu threshold [6,7,[12], [13], [14], [15], [16], [17]], the scanning electron microscope (SEM) assist threshold [5,[18], [19], [20]], and the porosity-adaptive threshold [21]. The Otsu threshold is the most widely used, but it is not always reliable [17]. Some findings indicate that the Otsu threshold overestimates the GDL's porosity compared to the porosity measured by mercury intrusion porosimetry (MIP) [21]. The SEM assist threshold uses SEM image information, such as fiber continuity and diameter, to determine the grayscale thresholds [[18], [19], [20]]. However, this process is generally empirical and may yield inaccurate results [18]. The porosity-adaptive threshold matches the model porosity with the experimental porosity measured by MIP. As MIP cannot experimentally measure some closed pores, the porosity-adaptive threshold may also deviate from the true value [22].

To address the issue of grayscale threshold selection, this study initially examines the impact of grayscale threshold variations on the morphology, mechanical, and transport properties of the reconstructed GDL. An X-ray CT scanner was used to scan the GDL sample. The experimental procedure is outlined in section 2. Section 3 discusses pre-processing tasks and the determination of the Otsu threshold and porosity-adaptive threshold. Lastly, we compare the Otsu and porosity-adaptive thresholds and analyze the sensitivity of porosity, tortuosity, permeability, and macroscopic stress-strain relationships to changes in grayscale thresholds. This study provides valuable references for the sensitivity of transport and mechanical properties to grayscale threshold variations of the reconstructed GDL and the selection of Otsu and porosity-adaptive threshold.

## 2 X-ray CT experiment

The GDL model used in this study is Toray carbon fiber paper (TGP-H-060) treated with 10 % polytetrafluoroethylene (PTFE), which lacks a microporous layer and is in an uncompressed state. The size of the scanned sample was X × Z = 2000 μm × 2000 μm. With reference to the study of Zhu [23] and Xiao [8,24], a region of 500 μm × 500 μm within the sample was selected for further analysis after the representative elementary volume (REV) size test.

X-ray CT scanning was performed using a Phoenix V|tome|x M300 scanner. X-ray CT rotates the sample progressively, capturing a tomographic image at a specific angle after each rotational step. These images are processed to generate a 3D model of the scanned part. The sample was affixed upright on the CT test bench. Scanning parameters were a voltage of 80 kV and current of 200 μA, yielding a resolution of 2 μm. It is worth noting that the grayscale distribution interval of the scanning results was in the range of 3297–7465.

## 3 Image processing

**Fig. 1:**
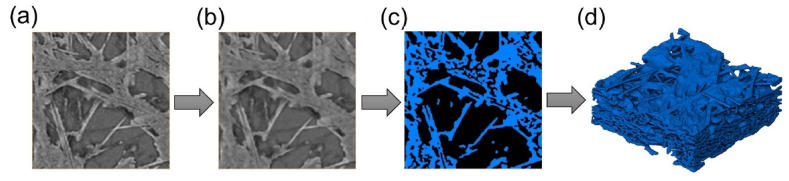
(a) An original grayscale image, (b) the image after median filter processing, (c) the binarized image (blue represents the solid domain, black represents the pore space), and (d) 3D digital model (solid domain is rendered). (For interpretation of the references to colour in this figure legend, the reader is referred to the Web version of this article.)

The original CT images were processed using Avizo to generate a 3D digital model. Initially, the original CT images as illustrated in Fig. 1(a),the 16-bit grayscale images, were filtered via a median filtering algorithm to eliminate noise, resulting in the images shown in Fig. 1(b). Median filtering involves a window size of 3 × 3 pixels, and the grayscale value at the central pixel is defined as the median of the grayscale values of these 9 pixels. The images then needed to be binarized, a critical step in GDL model generation. For a given grayscale threshold, voxels with grayscale values less than the grayscale threshold were defined as 0, representing the pore space, while voxels with grayscale values higher than the grayscale threshold were defined as 1, representing the solid space as shown in Fig. 1(c). Consequently, the model's porosity can be calculated as follows:

(1) $$\varepsilon =1-\frac{Entity\;voxels}{Total\;GDL\;voxels}$$

**Fig. 2:**
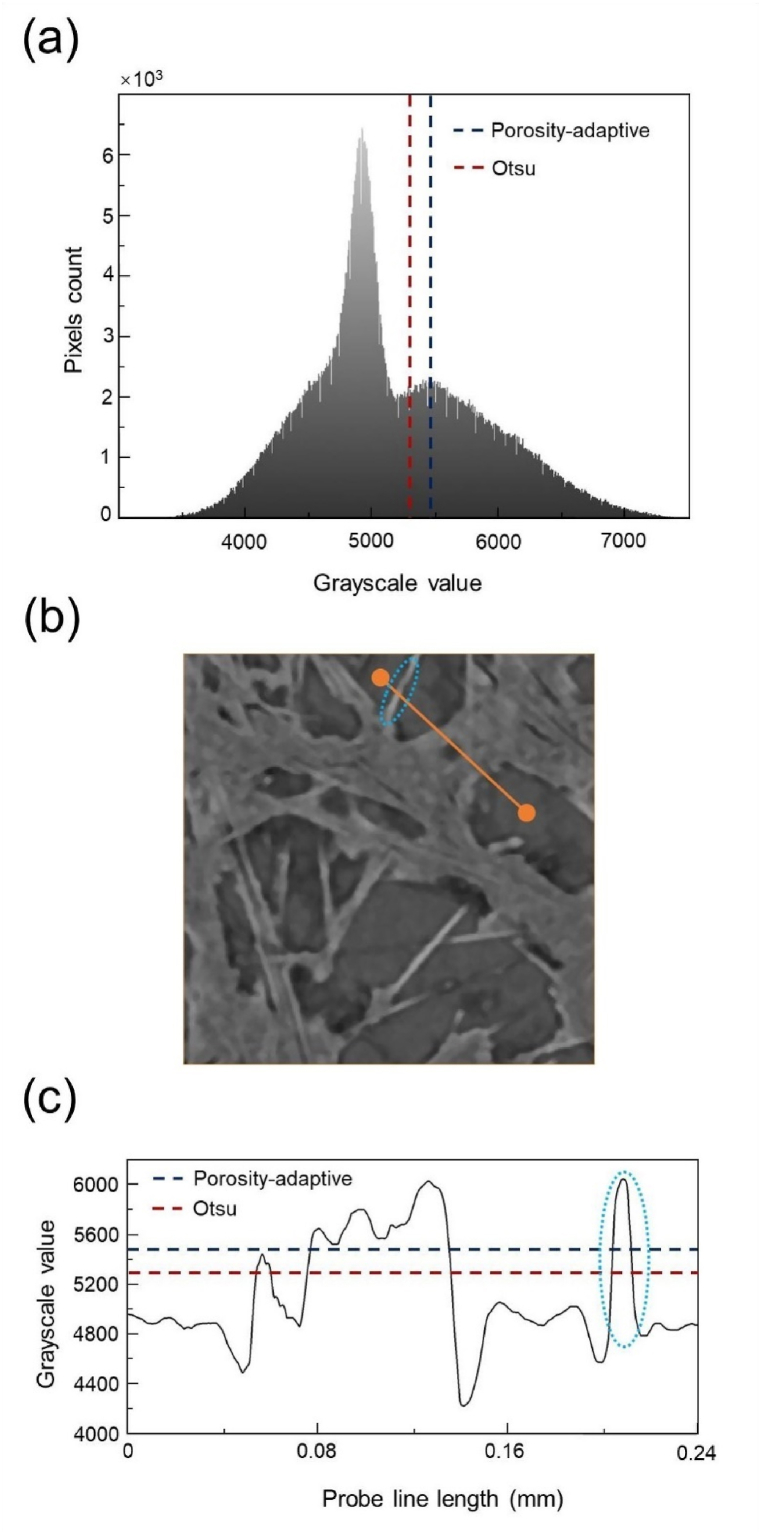
(a) Grayscale histogram of all data, (b) probe line on the CT image, and (c) grayscale distribution on the probe line.

The calculated porosity can serve as a reference index for determining the porosity-adaptive threshold. Notably, carbon fiber paper is usually treated with the addition of PTFE, and this PTFE content can affect the characteristics of GDLs [25]. But it is challenging to separate them using X-ray CT to construct an accurately detailed 3D model of the GDL. Thus, in similar studies, no distinctions were made between them, only between the solid region and the air [2,11,26]. Consequently, based on the porosity of the c,arbon fiber matrix, the porosity after PTFE treatment is obtained using the following equation:

(2) $$\varepsilon_{PTFE}=\varepsilon_{0}-a\frac{\omega \left(1-\varepsilon \right)}{1-\omega }$$

where $\varepsilon_{0}$ is the porosity of the carbon fiber matrix (78 %, as reported by Ref. [27]), and ω is the PTFE content, i.e., 10 %. Hao and Cheng proposed a=0.9 as the density ratio of the carbon fiber and PTFE [28]. The $\varepsilon_{PTFE}$ was calculated to be 75 %. By adjusting the grayscale threshold so that the values of ε and $\varepsilon_{PTFE}$ were equal, the porosity-adaptive threshold was calculated to be 5,486, as shown in Fig. 2. To give a clear impression of the porosity-adaptive threshold, it is marked in the grayscale histogram of all data in Fig. 2(a) and in the grayscale distribution along a probe line in Fig. 2(c), respectively. The peak marked in Fig. 2(c) corresponds to the fiber marked in Fig. 2(b).

The value of the Otsu threshold hinges on the maximum between-class variance, which can be calculated based on the image's grayscale information. The between-class variance represents the difference between the background and target grayscale values. The grayscale threshold at which this difference reaches its maximum is defined as the Otsu threshold. The between-class variance can be expressed as follows:

(3) $$\sigma^{2}\left(k\right)=\frac{{\left[\mu_{T}\cdot \omega \left(k\right)-\mu \left(k\right)\right]}^{2}}{\omega \left(k\right)\cdot \left[1-\omega \left(k\right)\right]}$$

where μ(k) is the expected value of the first k levels of grayscale in the total gray level L, $\mu_{T}$ is the global average of the image grayscale values, and ω(k) is the sum of the local frequencies of the first k levels of the grayscale values. These are given by:

(4) $$\mu_{T}=\mu \left(L\right)=\sum_{i=1}^{L}i\cdot p\left(i\right)$$

(5) $$\omega \left(k\right)=\sum_{i=1}^{k}p\left(i\right)$$

where p(i) is the frequency of occurrence of a particular grayscale value i, defined as the quotient of the number of pixels of grayscale value i to the total number of pixels:

(6) $$p\left(i\right)=\frac{n_{i}}{N}$$

in this manner, the Otsu threshold corresponding to the maximum between-class variance can be calculated:

(7) $$T=argmax_{k}\sigma^{2}\left(k\right)$$

For the sample in this study, the Otsu threshold was calculated to be 5,297, as illustrated in Fig. 2. It should be noted that these thresholds are not universal among different equipment, and depend on various factors such as X-ray energy, exposure time, magnification etc. In the subsequent study, a series of grayscale threshold points near the Otsu and porosity-adaptive thresholds are selected to investigate the effect of grayscale threshold variation on the mechanical and transport properties of the GDLs.

### 3.1 Characterization of transport properties

To analyze the impact of the grayscale threshold on the GDL transport characteristics, this study reconstructed the 3D digital model under various thresholds based on the CT images obtained from the experiment outlined in section 2. The effects of the threshold on tortuosity and permeability were assessed, with both parameters derived by solving the flow field in the pore space.

**Fig. 3:**
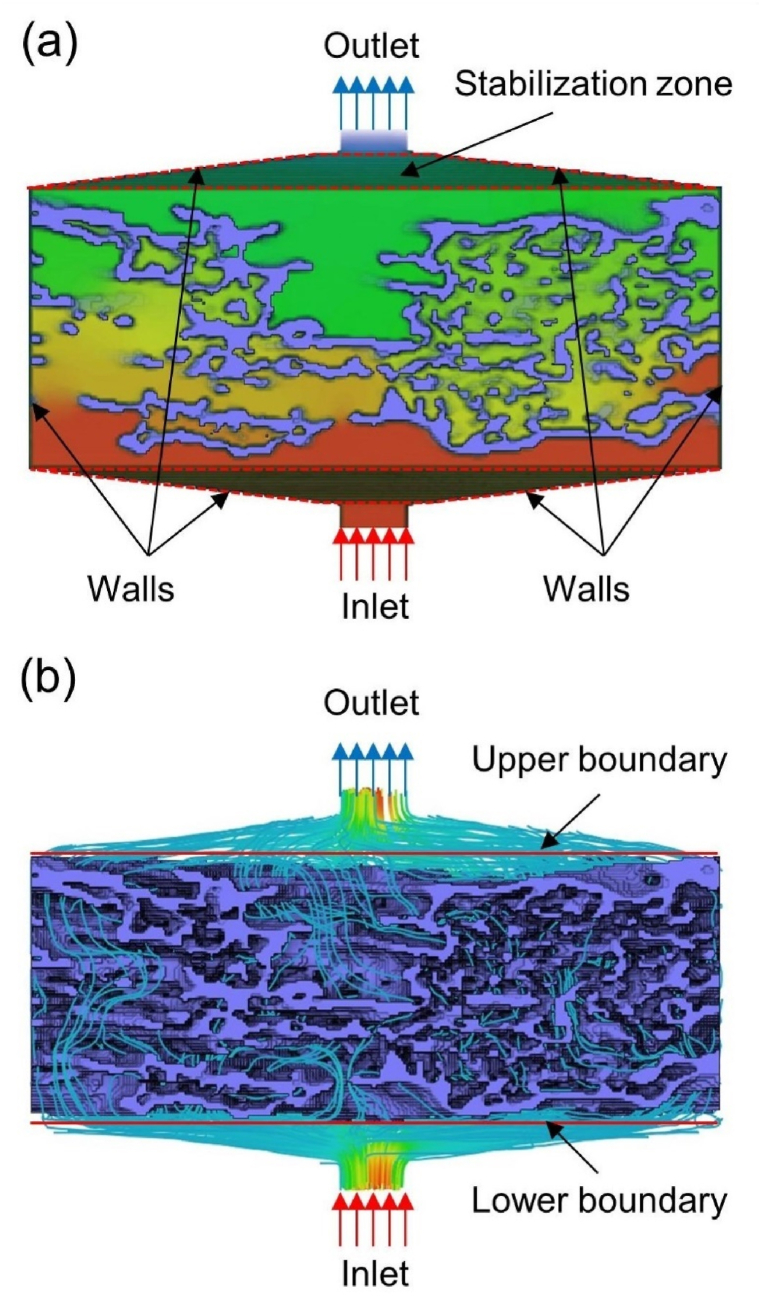
Boundary conditions and the solution of (a) pressure field and (b) velocity field in the TP direction for GDLs.

Assuming that the fluid is incompressible with constant density, the continuity equation and Navier-Stokes equation are as follows:

(8) ∇u=0

(9) $$\rho u\cdot \nabla u=-\nabla P+\nabla \hat{\tau }$$

where u is the velocity, ρ is the density, P is the pressure field, and $\hat{\tau }$ is the stress tensor. Based on the 3D numerical model, the above mass and momentum equations were solved and the permeability of the GDL was calculated using Darcy's law, as follows:

(10) $$V=-\frac{K}{\eta }\nabla P$$

where K is the permeability, V is the superficial velocity (i.e., volumetric flow rate per unit cross-sectional area), η is fluid viscosity, and ∇P is the pressure drop per unit length through the GDL. For the simulation, the pressure field as shown in Fig. 3(a) and velocity field as shown in Fig. 3(b) are solved by the finite volume method (FVM) in Avizo X-Lab. The boundary conditions are shown in Fig. 3. The inlet pressure is set to 100 Pa and the outlet pressure to 0 Pa in both through-plane (TP) and in-plane (IP) directions. The fluid viscosity is 0.002 Pa s and non-slip boundary conditions are applied to the wall of fluid simulation. The stabilization zone where pressure is quasi static can make the fluid freely spread on the upper and lower boundaries of the GDL vertical to the direction of flow,i.e., the TP direction in Fig. 3.

The expression of tortuosity (τ) is shown in Eq. (11):

(11) $$\tau =\frac{\frac{1}{N}\sum_{i=1}^{N}l\left(r_{i}\right)}{L}$$

where N is the number of traced fluid particles and $l\left(r_{i}\right)$ is the length of the path line belonging to the i th particle located at $r_{i}$. L is the characteristic length of the porous medium in the flow direction, i.e., the thickness of the GDL in the TP direction and the width in the IP direction in this case. The flow path for each particle (e.g., the streamline) is generated by the vector field, and the tortuosity can be obtained by calculating Eq. (11). The results of tortuosity are based on 224 and 525 streamlines in TP and IP directions respectively to ensure the accuracy. Tortuosity defines the ratio of the average length of flow paths to the characteristic length of the GDL [29]; it represents the complexity of the microfluidic paths within the GDL. Therefore, it serves as an important indicator of transport performance.

### 3.2 Solid mechanics simulation

**Fig. 4:**
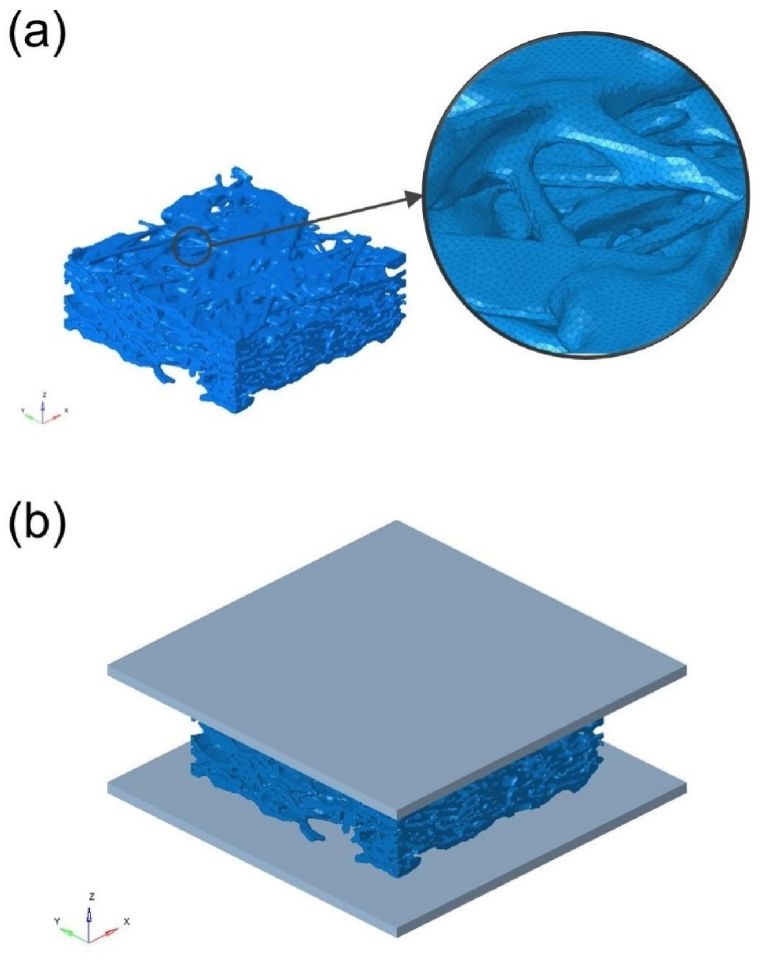
(a) The GDL model after proper smoothing and meshing, and (b) solid mechanics simulation assembly.

The smoothing and meshing of the GDL model as shown in Fig. 4(a), were carried out in Avizo and Hypermesh respectively, using the same smoothing parameters and element size of 3 μm. The solid mechanics simulation was performed using Abaqus software, taking into account the dynamic contact, compression, and bending of the carbon fibers in the GDL model. The GDL was assembled between two rigid plates, with a displacement load applied to the upper plate while the lower plate was fixed, thus compressing the GDL as shown in Fig. 4(b). The large deformation theory was considered in the simulations. A uniform material property was assigned to the GDL model [27], with Young's modulus set at 6000 MPa and a Poisson ratio of 0.256. Furthermore, tangential friction between fibers was ignored, with the normal direction defined as hard contact. The contact conditions are performed by penalty function method. The above-mentioned simulation parameters ensure that the rigidity of the GDL model (at the threshold 5540) in a 30 % compression ratio state aligns with the experimental values [27], i.e., the ratio of force to displacement is 5.8 N/mm.

Following the simulation, the forces and displacements were converted into stresses and strains for macroscopic GDLs to facilitate analysis:

(12) $$\sigma_{macro}=\frac{F}{A}$$

(13) $$\varepsilon_{macro}=\frac{U}{L}$$

where $\sigma_{macro}$ represents the calculated macroscopic stress, F is the magnitude of the concentrated force exerted on the upper surface, and A is the GDL area, $\varepsilon_{macro}$ signifies the macroscopic strain, U is the displacement of the upper surface, and L represents the GDL thickness.

## 4 Results and discussion

In this section, we examine a series of grayscale threshold points near the Otsu and porosity adaptive thresholds to investigate how grayscale threshold variation impacts the morphological, mechanical, and transport properties of GDLs. The grayscale threshold interval containing 10 thresholds (i.e., 5297, 5324, 5351, 5378, 5405, 5432, 5459, 5486, 5513 and 5540) is defined as the study interval. It should be noted that the following results are based on PTFE-treated samples. The PTFE-untreated samples may be different.

### 4.1 Effect of grayscale threshold on morphology

**Fig. 5:**
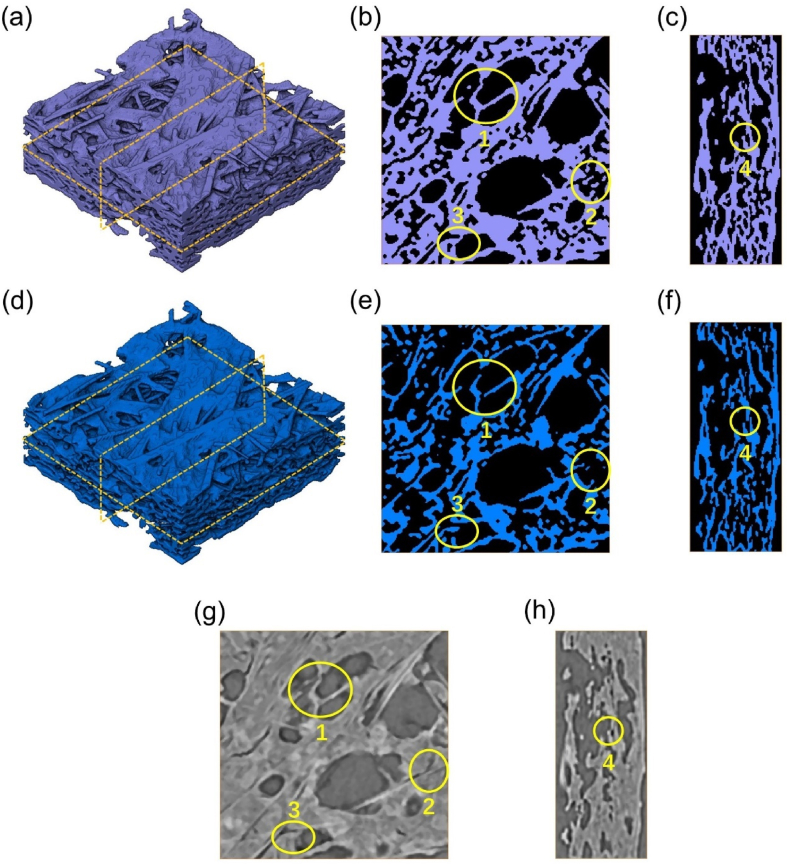
(a) GDL structure and slices in the (b) TP and (c) IP directions under the Otsu threshold, (d) GDL structure and slices in the (e) TP and (f) IP directions under the porosity-adaptive threshold, and relevant grayscale images in the (g) TP and (h) IP directions.

Fig. 5 illustrates the 3D structure of the GDL model under both the Otsu and porosity-adaptive thresholds. It is evident that the difference between these two thresholds significantly impacts the structure and morphology of the GDL. As highlighted in Fig. 5(e), some structural connections appear broken in zone 1 and zone 3 at the porosity-adaptive threshold. This phenomenon can also be seen in the IP direction from zone 4 in Fig. 5(f). The breaks of such connections can cause a change in the flow path structure. Furthermore, the volume of irregular PTFE is also markedly diminished in area 2 at the porosity-adaptive threshold in Fig. 5(e). In summary, we can see from the slices that the grayscale threshold variation brings a considerable change to the structure and morphology of the GDL.

### 4.2 Effect of grayscale threshold on porosity

**Fig. 6:**
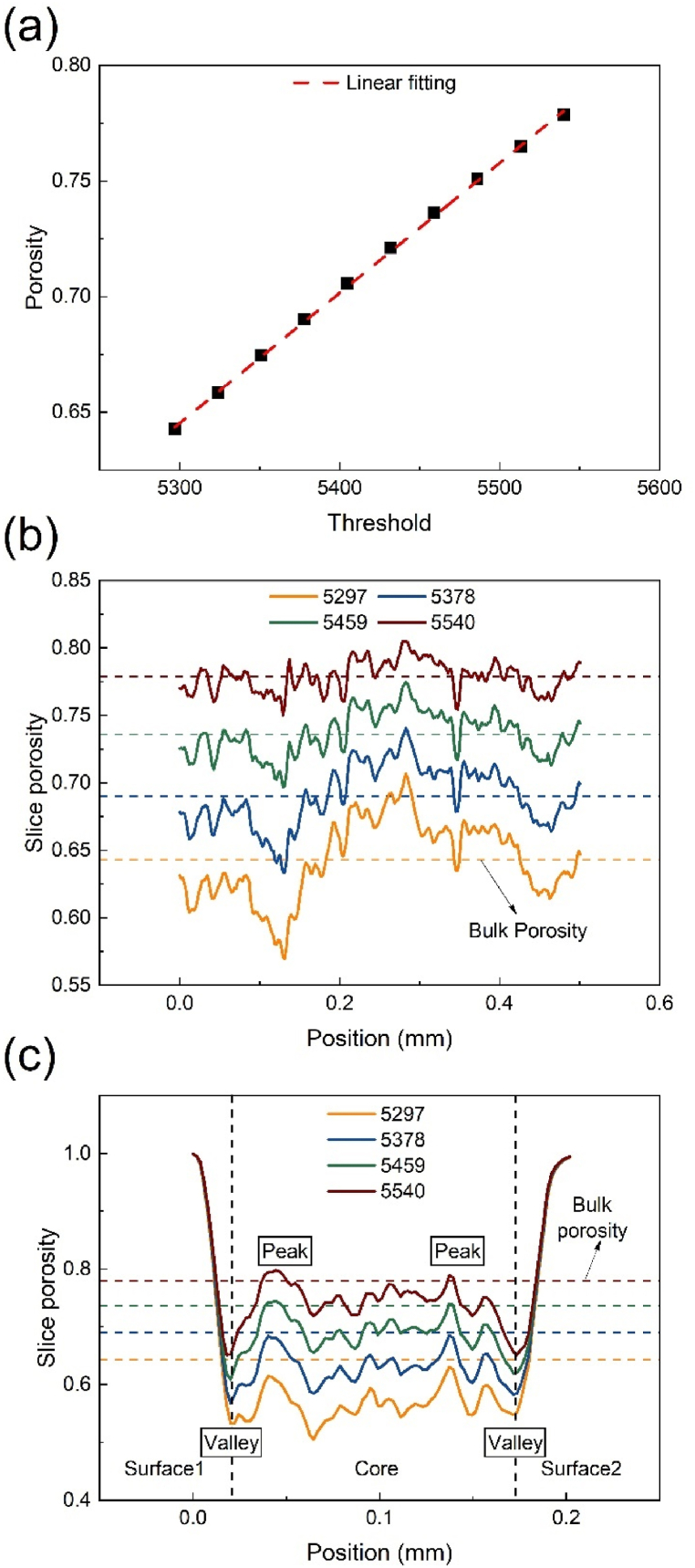
(a) Bulk porosity under different grayscale thresholds, and slice porosity under different grayscale thresholds in (b) the IP direction and (c) the TP direction.

The bulk porosity was assessed for 10 sets of grayscale thresholds situated near the Otsu threshold (5,297) and the porosity-adaptive threshold (5,486). Fig. 6(a) illustrates the curve of the bulk porosity change in relation to the grayscale threshold. The results suggested that porosity varies approximately linearly with the grayscale threshold, with a 1 % alteration in grayscale threshold yielding a 4 % modification in porosity. In comparison to the porosity determined by Eq. (2), the bulk porosity under the Otsu threshold is underestimated by 10 %.

In the study of GDL slice porosity, changes in porosity are compared at four grayscale thresholds (5,297, 5,378, 5,459, and 5540), as depicted in Fig. 6(b) and (c). In the IP direction, the slice porosity of the GDLs fluctuates randomly around the bulk porosity in Fig. 6(b). The slice porosity fluctuation in high-level grayscale thresholds is significantly smaller than that in low-level grayscale thresholds. We can thus conclude that the random variation in slice porosity diminishes as the grayscale threshold escalates. A similar phenomenon is observed in the TP direction in Fig. 6(c), where the porosity distribution is split into two transitional surface areas and a core region, consistent with the findings of Fishman [14]. Furthermore, the grayscale threshold has little effect on the porosity of the surface regions, but markedly influences the porosity of the core region. At the junction of the surface and core regions, where the two valleys are located, the porosity is low, while porosity peaks near the edge of the core region. This shows that the stacking layers utilized to fabricate Toray GDLs may consist of multiple TGP-H-030 layers [30,31]. The observed porosity distribution pattern is attributed to the multiple layer compression steps undertaken during GDL manufacturing.

### 4.3 Effect of grayscale threshold on tortuosity and permeability

**Fig. 7:**
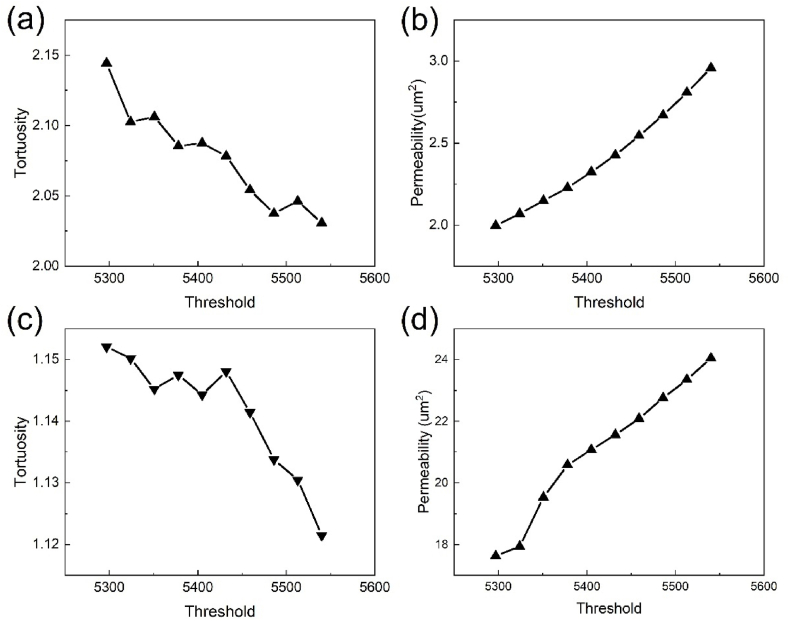
(a) Tortuosity and (b) permeability in the TP direction under different thresholds, and (c) tortuosity and (d) permeability in the IP direction under different thresholds.

The parameters of tortuosity and permeability are obtained via computational fluid dynamics simulations in the TP direction. Tortuosity exhibits a general downward trend with the increase of grayscale threshold, yet it does not change monotonically with the variations in grayscale threshold from Fig. 7(a). As grayscale thresholds increase, changes occur in the flow channel structure and size within the pore space, leading to minor fluctuations in tortuosity. Overall, within the study interval, every 1 % change in grayscale threshold results in an average change of 1.3 % in tortuosity. Permeability rises with the increasing grayscale threshold, with an average increase of 7.4 % in permeability for every 1 % change in grayscale threshold in Fig. 7(b). In comparison with the parameters under the porosity-adaptive threshold, the tortuosity at the Otsu threshold is overestimated by 5.2 %, while permeability is underestimated by 25.2 %.

In the IP direction, tortuosity also exhibits a downward trend with increasing grayscale thresholds, yet does not decrease monotonically in Fig. 7(c). Tortuosity appears more sensitive to alterations at higher grayscale thresholds and every 1 % change in grayscale threshold results in an average change of 0.7 % in tortuosity. Permeability exhibits a monotonic rise with increasing grayscale thresholds, showing an average increase of 6.1 % in permeability for every 1 % change in grayscale threshold within the study interval in Fig. 7(d). When compared with parameters under the porosity-adaptive threshold, tortuosity at the Otsu threshold is overestimated by 1.6 % and permeability is underestimated by 22.5 %.

Unlike the TP direction, IP tortuosity exhibits greater sensitivity to changes in higher grayscale thresholds. Through the Eq. (11) for tortuosity, we can conclude that the tortuosity is significantly influenced by the flow channel structure which includes degree of bending and the number of flow paths. Thus, it can be inferred that major alterations occur in the flow channel structure in the IP direction at higher grayscale thresholds. By comparing the flow parameter changes in both directions, we find that tortuosity in the IP direction is smaller than in the TP direction. At the same threshold, the IP tortuosity is approximately half of the TP tortuosity. This supports the assertion that the fiber orientation is largely along the IP direction. High tortuosity tends to result in low permeability. Therefore, the IP permeability significantly surpasses the TP permeability.

### 4.4 Macroscopic compressive mechanical response

**Fig. 8:**
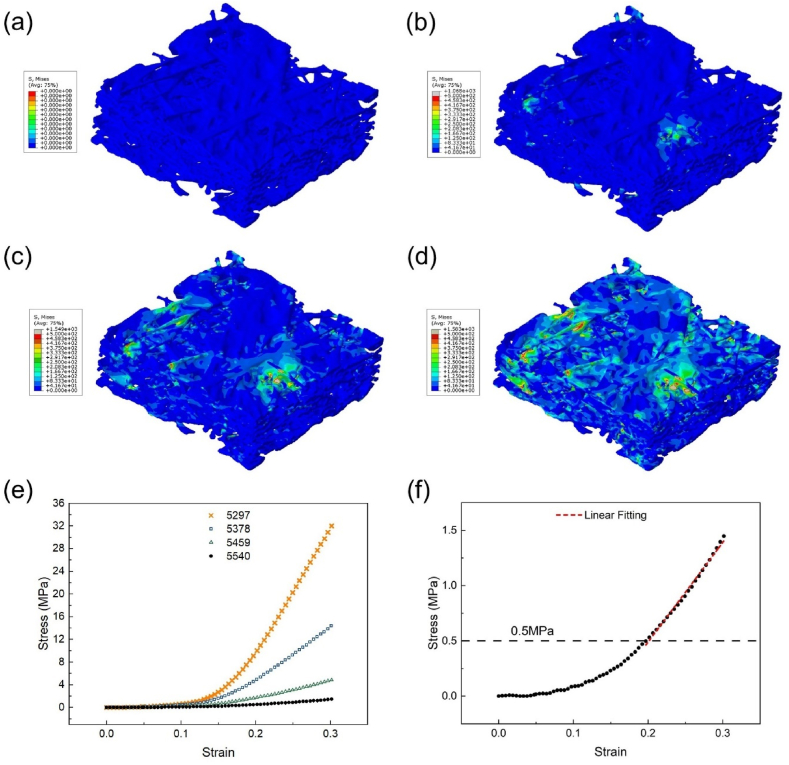
Compression models of the GDL at (a) 0 %, (b) 10 %, (c) 20 % and (d) 30 % compression ratio under the grayscale threshold value 5,540, (e) the macroscopic stress-strain relationship under four different thresholds, and (f) the stress-strain data under the grayscale threshold value 5540.

Further solid mechanics simulations are performed on four threshold values (5,297, 5,378, 5459 and 5540) within the study interval. As an example, the compression process of the GDL model at the grayscale threshold value 5540 is give in Fig. 8. This figure demonstrate that it is possible to conduct structural mechanics simulation based on the reconstructed GDL model, which has not been conducted by other researchers before. This simulation method has a potential application for investigating the effect of assembly pressure on the performance of a PEM fuel cell. In the current study, we focus on the effect of grayscale threshold on the macroscopic stress-strain relationship of the GDL, as given in Fig. 8(e). The results reveal that even minor changes in the grayscale threshold result in significant differences in the GDL's mechanical properties. As strain increases, the stress differences between models corresponding to different grayscale thresholds become increasingly pronounced, indicating substantial disparity in stresses associated with models at different thresholds under high strain conditions.

Taking the stress-strain data under grayscale threshold 5540 as an example, the stress-strain relationship appears to be roughly exponential Fig. 8(f). This suggests an increasingly large contact area between the fibers. The pressure 0.5 MPa as marked in Fig. 8(f) represents a typical pressure used to make the membrane electrode assembly (MEA) [32,33]. Therefore, the region above 0.5 MPa is the primary focus of this study, and a rough linear fitting is used to determine the equivalent macroscopic elastic modulus ($E_{macro}$). The values of $E_{macro}$ at the four grayscale thresholds are 221, 94, 31, and 9 MPa (with a corresponding grayscale threshold from low to high). $E_{macro}$ is highly sensitive to changes in grayscale threshold, with $E_{macro}$ at the grayscale threshold 5540 being just 4 % of that at 5297. On average, a 1 % change in the grayscale threshold results in a 21.8 % change in $E_{macro}$.

### 4.5 Discussion of the two thresholds

A comparison of the above results reveals a significant difference between the Otsu threshold and the porosity-adaptive threshold. As illustrated in Fig. 2(a), the two peaks of the grayscale histogram theoretically represent the two different densities of material in the scanned domain. The capabilities of our equipment (Phoenix V|tome|x M300) may still not be sufficient to distinguish between carbon fibers and PTFE accurately. And it is the mostly likely reason for only two peaks on the grayscale histogram.

A reasonable grayscale threshold should be located in the region between these two peaks. Therefore, the porosity-adaptive threshold appears to be less accurate than the Otsu threshold, possibly because the porosity value (75 %) we used was inaccurate. We believe the porosity of the TGP-H-060 with PTFE is not a constant. It not only depends on the content of PTFE, but also the manufacturing process. 75 % is only an estimated value based on the experimental results from other researchers [27,28]. The porosity value may vary apparently among different GDL samples, even if the sample has the same content of PTFE. It is difficult for researchers to obtain the exact porosity value for each specific GDL sample before X-ray CT reconstruction. The grayscale threshold based on the estimated porosity value may deviate from the accurate value apparently. For this reason, we suggest that the Otsu threshold is more suitable for related studies, as it can circumvent issues with porosity inaccuracy arising from sample variability.

## 5 Conclusions

In recent years, X-ray CT has been used by some scholars to reconstruct 3D models of GDLs to study the transport phenomena of GDLs. In the process of reconstructing 3D models of GDLs based on X-ray CT, the selection of grayscale thresholds will directly affect the accuracy of the reconstructed GDLs. However, most scholars ignored discussing this issue. In this paper, Phoenix V|tome|x M300 scanner was used to scan and reconstruct a TGP-H-060 sample. Subsequently, the effects of grayscale threshold on the morphology, permeability, tortuosity and compressive mechanical properties were investigated. The results showed that the grayscale threshold had a significant effect on the reconstruction accuracy of the GDLs. Specifically, each 1 % change in grayscale threshold resulted in an average change of 4 % in porosity, 7.4 % in TP permeability, 6.1 % in IP permeability, 1.3 % in TP tortuosity, 0.7 % in IP tortuosity and 21.8 % in macroscopic elastic modulus.

We also compared and analyzed two grayscale thresholds commonly used by scholars, the Otsu threshold and the porosity-adaptive threshold. It is found that the porosity-adaptive threshold is clearly not accurate in our study, while the Otsu threshold is more reasonable. We attribute this difference to the inaccurate porosity values. Since it is often difficult to obtain the exact porosity value of each sample in advance, we suggest that Otsu threshold should be used in relevant studies.

## Data availability statement

The data that support the findings of this study are available upon reasonable request from the author. Researchers interested in accessing the data can contact Xiaoyu Ding email at xiaoyu.ding@bit.edu.cn.

## Additional information

**Fig. 9:**
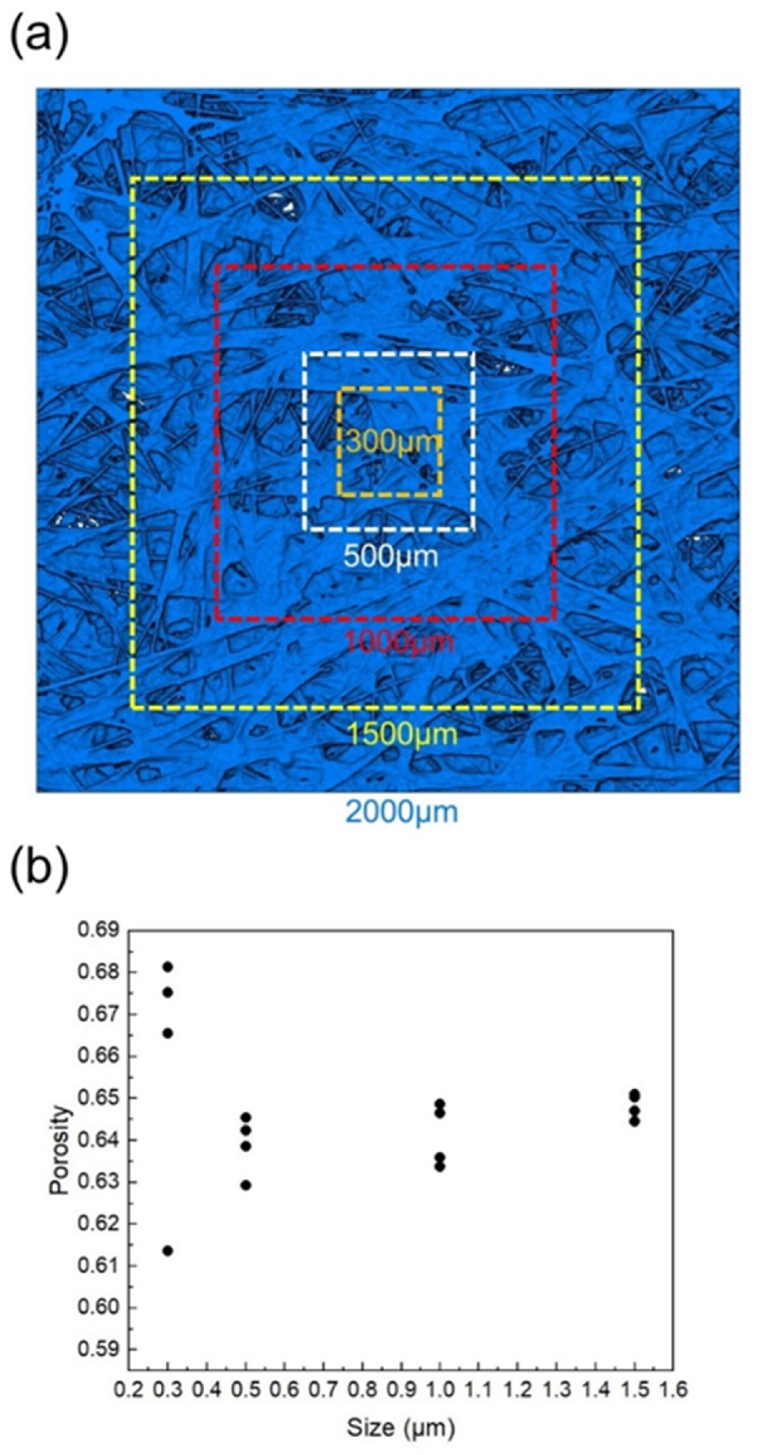
(a) The selected different sizes of REV, and (b) porosity analysis with different REV sizes.

In this study, the REV was selected according to the work of Zhu [23] and Xiao [8,24]. Meanwhile, in order to explain the representativeness of REV and add further strength to this study, we carried out porosity analysis of samples of different REV sizes which were 1500 μm, 1000 μm, 500 μm and 300 μm as shown in Fig. 9(a). The samples were reconstructed using the same image processing method as shown in section 3, and Otsu threshold was adopted in the analysis. For each specified size, we randomly selected four samples and analyze the porosities as shown in Fig. 9(b). When the REV size was less than 500 μm, the variance of porosity increased significantly with the decrease of REV size. We concluded that the REV of 500 μm × 500 μm in this sample is enough to achieve high accuracy of calculation, and the calculation time was greatly reduced compared with larger REV sizes.

## CRediT authorship contribution statement

**Huarui Li:** Visualization, Validation, Software, Methodology, Investigation, Formal analysis, Data curation, Conceptualization. **Tingqiang Qiao:** Supervision, Resources, Project administration. **Xiaoyu Ding:** Writing – review & editing, Supervision, Project administration, Funding acquisition, Conceptualization.

## Declaration of competing interest

The authors declare that they have no known competing financial interests or personal relationships that could have appeared to influence the work reported in this paper.
